# First physical evidence for forested environment in the Arctic during MIS 3

**DOI:** 10.1038/srep29054

**Published:** 2016-07-01

**Authors:** Pertti Sarala, Minna Väliranta, Tiina Eskola, Giedré Vaikutiené

**Affiliations:** 1 Geological Survey of Finland, P.O. Box 77, FI-96101 Rovaniemi, Finland; 2 Department of Environmental Sciences, University of Helsinki, P.O. Box 65, FI-00014, Finland; 3 Oulu Mining School, University of Oulu, P.O. Box 3000, FI-90014, Finland; 4 Vilnius University, Čiurlionio 21/27, LT-03101 Vilnius, Lithuania

## Abstract

Old sedimentological and geochronological records can be preserved underneath the central parts of the continental ice sheets under non-erosive, cold-based subglacial conditions. Organic deposits that predate the last deglaciation are of particular value for the information held on glacial-time climate and environmental conditions. In this study, we present multiproxy data derived from a well-preserved MIS 3 interstadial (55–25 ka ago) organic layer from inside the Arctic Circle in the Finnish Lapland. Biological proxy evidence, namely coming from aquatic plant species, indicates July temperatures as high as 14.4 °C, i.e. higher than those of today for the study site. Macrofossil evidence demonstrates for the first time the presence of pines accompanied by tree birch during the MIS 3 interstadial in northern Fennoscandia. These results concur with contemporary insolation model outcomes but contradict with the previous proxy-based view of open tundra conditions during the MIS 3. The data suggest that there are highly dynamic interstadial continental ice-sheet dynamics following changes in orbital forcing. Warm climate enabled the establishment of forests on exposed landscape. Moreover, we suggest that in the light of these new data, previous MIS 3 pollen data could be re-interpreted.

Undisturbed interstadial organic layers are rare in glaciated regions although in subglacial, cold-based conditions old sediment deposits are commonly found^1^,^2^,^3^,^4^. Despite the Marine Isotope Stage 3, MIS 3 (55–25 ka ago) sediment sections in northern Fennoscandia are scattered and fragmented, and the chronological control of these layers have proven challenging, and the site-to-site comparison is complicated because the sediment varies from peat to highly minerogenic, the existent of ice-free MIS 3 conditions is not a debatable issue anymore. Study sites with robustly dated sediment sections that testify ice-free conditions span over whole Fennoscandia and some of these records also include, not only dated organic sediment layers, but also palaeobotanical data which demonstrate interstadial succession of vegetation^5^,^6^,^7^,^8^,^9^,^10^,^11^,^12^,^13^. This interpretation is supported by insolation models which suggest that in northern Scandinavia the insolation deviation during the MIS 3 period was positive, i.e. summer temperatures may have been even slightly higher than today but lower than for instance during the MIS 5 e and c^14^. MIS 5 intervals with higher insolation has been reflected in palaeobotanical records as a more northern distributions of many plant species that currently occur in south or middle boreal zone^11^,^15^,^16^. Yet, previous MIS 3 proxy data have not lent support to a forested northern Fennoscandian environment, while in Denmark the macrofossil records testify the presence of trees and also other boreal plant species^17^. By contrast for northern Fennoscandia an open tundra environment has been suggested. However, it should be noted that previous interpretations have mainly based on percentage pollen data^1^,^18^,^19^,^20^,^21^ and because often the establishment of a proper age-depth model is hindered, pollen accumulation rate calculations cannot be implemented, and thus pollen data are not suitable to infer the presence of forests. To date for northern Fennoscandia macrofossil data have been scarcely available^2^,^12^,^22^,^23^,^24^,^25^,^26^,^27^. Macroscopic remains can provide invaluable evidence of actual presence of species, while pollen records also reflect long-distance transportation. Recent advancement in Optically Stimulated Luminescence (OSL) dating techniques to date sediments older than ca. 50 000 years^28^,^29^, which is the age limit for radiocarbon ^14^C method^30^, has increased the array of geochronological tools to investigate old inter-till stratified deposits. Here, we present palaeoecological data derived from a MIS 3 section collected from Kaarreoja (Fig. 1) in Finnish Lapland. By applying OSL and ^14^C datings and multiproxy approach, we show the first evidence of presence of pine and birch forest accompanied by other boreal species in northern Fennoscandia during the MIS 3. We use macrofossils to quantitatively reconstruct MIS 3 temperatures as performed in Väliranta *et al*.^31^. Moreover, we aim to provide an insight how the MIS 3 percentage pollen diagrams could be reinterpreted.

## Results and Interpretation

### Sediment stratigraphy

The whole exposed section POS$-2012-29 includes a full stratigraphical history from the Middle Weichselian stadial (MIS 4) to the present day (Fig. 2). The sequence starts with the glacial advance indicated by diamicton till in Unit 1. Unit 1 is followed by glaciofluvial gravel sediments in Unit 2, this demarks deglaciation. Unit 3 is characterised by glaciofluvial/fluvial sands and silts (see Supplementary Fig. S1) and indicates flowing water activity which turns into a sluggish flow phase in the upper part of the unit. Littoral organic sediments (gyttja) compose Unit 4 suggesting a formation of a lake or pond. The Unit 5 is comprised of organic peaty deposit with abundant wood remains. Above this organic layer, there is a hiatus caused by the next glacial advance. The sedimentary section continues as a layer of glaciofluvial and fluvial sands with some pebbles (Unit 6). Above that is a massive diamicton unit (till) representing the latest glacial advance phase in the area (Unit 7). The till deposit is overlain by glaciofluvial deposit which is gradually changing into modern fluvial deposits on top (Unit 8). On the surface, gravelly sands are mixed with organic material (Unit 9).

### Chronology

**Figure 1:**
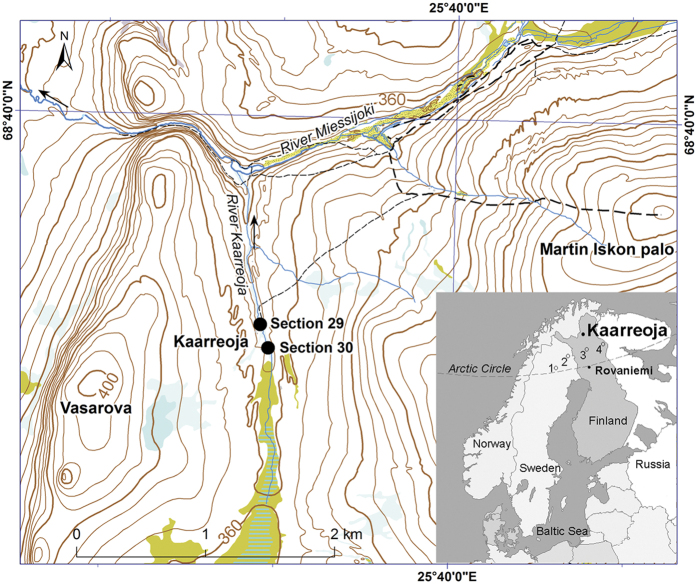
Location of the Kaarreoja study area and the sampling sites in northern Finland. Known MIS 3 organic deposits inside the Arctic Circle in Scandinavia are also marked in the map, 1) Rissejauratj, 2) Riipiharju, 3) Petäjäselkä and 4) Sokli. Coloured shadings mean peat covered areas and turquoise coloured lines indicate open peatland areas. Geographic coordinate system: EUREFFIN. Contains data from the National Land Survey of Finland Topographic Database 03/2015 (National Land Survey open data CC 4.0 licence) and MapsOpenSource (a Creative Commons Attribution 3.0 Unported License).

**Figure 2:**
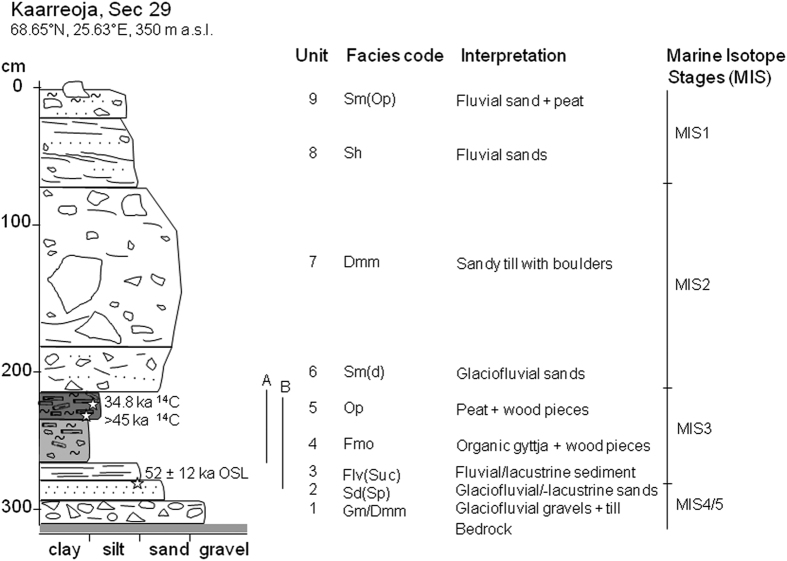
Stratigraphy log of the test pit POS$-2012-29 including the sampling sites of organic material and dating samples. The MIS 3 organic deposit is located within sandy/gravelly layers of which the lower was dated 52 ± 12 ka by OSL dating method. Line A = continuous sediment section for the pollen and macrofossil analyses and line B = continuous sediment section for the diatom analyse.

Two fine sand samples were dated by the Optical Stimulated Luminescence (OSL) method. The sample from the section POS$-2012-30 (see Fig. 1) underlay thick till (comparable with Unit 1 in section POS$-2012-29) and yielded an age of 133 ± 28 ka while the other OSL sample from the Unit 3 in the section POS$-2012-29 (Fig. 2) which underlay the organic sediment section yielded an age of 52 ± 12 ka (Supplementary Table S1). The consistency of data (8 and 5 aliquots) was good ranging within 2 sigma variation (Supplementary Fig. S2). The relatively large age error ranges are typical for OSL-determined ages^32^ and this reflects the fact that samples also contain some older, incompletely bleached minerogenic material. To evaluate the reliability of the OSL dates we radiocarbon dated one bulk peat sample from the overlying organic layer. The uncalibrated age of the bulk peat sample was 30,200 ± 250 years BP (34,800 ± 180 years BP cal based on Reimer *et al*.^33^). Moreover, wood from the same organic layer but below the bulk peat sample yielded an AMS ^14^C age of >45,000 years BP. These results suggest that despite some indication of fluvial disturbance, layers are in chronological order and the studied organic-bearing section (Units 3–5) represent MIS 3 (55–25 ka ago). The Units 1 and 2 represents an earlier cold stage, most probably MIS 4 and the Units 6 and 7 deposited during MIS 2 period.

### Pollen assemblages

**Figure 3:**
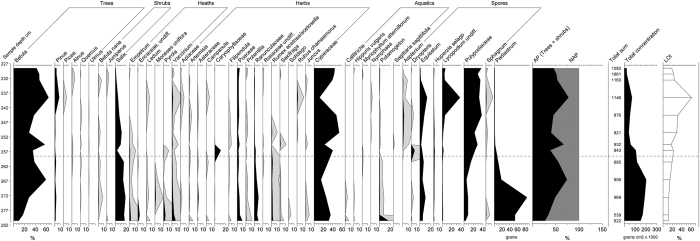
Selected pollen diagram. Hollow curves correspond with an exaggeration x10.

*Betula* is the dominant tree pollen and the amount varies from 8 to 65% (Fig. 3). *Pinus* pollen is present in rather small quantities, from less than 5% to maximum of 8%. *Alnus* and thermophilous trees such as *Quercus* and *Ulmus* occur at a low percentage in a few samples and probably indicate a long-distance transportation. Also, a well preserved pollen grain of *Picea* was identified in one sample. *Salix* is constantly present but decreases towards the top of the unit. The highest levels of dwarf-shrubs such as *Vaccinium*, *Empetrum* and *Pyrola* are found at the bottommost samples. Poaceae, Cyperaceae, Ranunculaceae and *Rumex acetosa/acetosella* are the major herb taxa. Spores of *Equisetum, Lycopodium* and Polypodiaceae are well-represented in every sample. Aquatic species such as *Callitriche*, *Potamogeton*, *Sagittaria sagitifolia*, *Hippuris vulgaris* and *Myriophyllum* occur mainly at the lowest section with algae spores of *Pediastrum*, while Nymphaeae pollen were found from the top part of the section. Overall, aquatic pollen represent local presence and the species assemblages suggest boreal zone conditions.

The pollen stratigraphy can be divided into two phases. First of all, proportional changes in aquatic species such as *Callitriche*, *Potamogeton*, *Hippuris vulgaris* and *Myriophyllum* and wetland taxa such as cryptogams, originating from local sources, indicate a landscape change where the open water deposition environment became more extensively surrounded by a wetland. Moreover, during the aquatic period, lower percentages of *Betula* and higher percentages of *Salix*, Ericaceae, Poaceae and Ranunculaceae indicate a more open environment. Then the open vegetation was replaced by a *Betula* forest with evidence of *Pinus* trees, as the nearly 10% of *Pinus* pollen indicate. This value is close to a threshold value 10% which is said to indicate regional presence of pine based on the comparison between modern pollen data and plant species distribution information^34^. Also modern pollen data collected from Finnish Lapland suggest that *Pinus* pollen values from 10 to 50% are an indication of local birch with pine vegetation^35^.

An increase in *Pinus* and thermophilous species percentages suggest a change to warmer regional-scale climate in the upper part of the sediment stratigraphy. However, thermophilous species probably merely reflect long-distance transportation or possibly re-deposited pollen.

### Macrofossil assemblages

**Figure 4:**
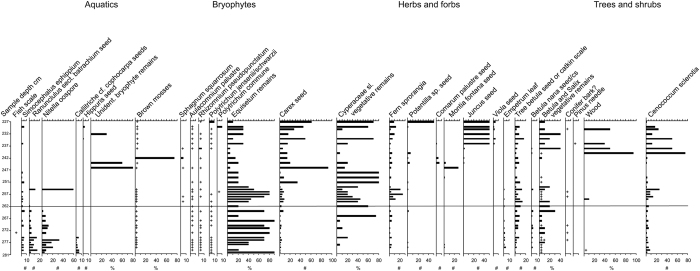
Macrofossil diagram. Black bars indicate number of finds and a symbol ‘+’ indicates the presence.

The macrofossil record can be divided into three phases: 1) a phase with the presence of aquatic plant species and fish from 281 cm to 262 cm. These samples also contained large amounts of *Equisetum* remains, sand and sometimes also stones and this indicates fluvial activity. Tree *Betula* seeds were continuously present already from the beginning suggesting presence of birch forest. Potentially also conifer remains were present at the lower section even though a small size and the high level of decomposition did not completely allow exhaustive identification of these (bark) remains. Small amounts of bryophytes were consistently detected. 2) a phase where aquatic species are not present but wetland species, such as ferns, Cyperaceae, *Comarum palustre*, *Montia fontana* and *Potentilla* sp. are abundant (depth 262–227 cm). The samples in this layer also contain mineral material but upwards the sediment became peatier. The analysed samples were characterized by presence of coarse organic material rather than presence of bryophytes which were however detected in small numbers until the top, 227 cm. Again, tree *Betula* seeds were present throughout. 3) a phase reflecting surrounding forested environment. The amount of wood was conspicuously high in the top samples (above 240 cm). One sample contained *Pinus* needle and this confirms presence of pine. Increase in *Cenococcum* sclerotia and *Polytrichum commune* corresponds with the large amount of wood and suggest drier forested terrestrial environment (Fig. 4).

### Diatom assemblages

**Figure 5:**
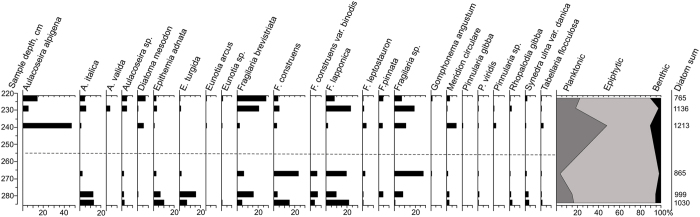
Diatom diagram shows selected species (which percentages are >0.5%) and some ecologically important taxa.

Six samples were analysed to supplement plant data. Numerous diatoms were found in sediment samples (counted more than 500–1000 diatoms per slide). Number of broken valves was very small. In total 53 diatom species were identified but only relevant selected taxa are presented (Fig. 5).

The lower part of the sequence (depth 285–255 cm) is characterized by freshwater epiphytic (attached mostly to aquatic plants) diatoms such as species from genera *Fragilaria* and *Epithemia* (*Fragilaria brevistriata, F. lapponica, F. cosntruens, F. construens. var binodis, Epithemia adnata, E. turgida*)^36^,^37^,^38^. These species have relatively high requirement of oxygen^36^. In the bottommost part of the section percentage share of planktonic diatoms is 17%. However, the most common *Aulacoseira italica* is described as planktonic-benthic in some sources^38^,^39^.

The diatom assemblages including reophilous taxa, such as *Meridion circulare* and *Diatoma mesodon* increase to upward (255–222 cm) in the sequence. These taxa thrive in running waters^38^,^40^. The most conspicuous change in the diatom record is the appearance and increase up to 50% of planktonic taxa (especially *Aulacoseira alpigena*) to the topmost samples. *Aulacoseira valida* found from one of the top samples is a species that can tolerate dystrophic, i.e. brown, waters with low pH^41^. An increased content of small planktonic *Aulacoseira* sp. taxa indicates increased nutrient enrichment and turbidity in the water basin^42^,^43^. Presence of reophilous diatoms also demonstrates the existence of fluvial environment, activity of flows near the investigated area. Establishment of forests as suggested by macrofossil and pollen data probably lead to increased leaching of humic substances to downstream waters.

## Discussion

The Kaarreoja section from Finnish Lapland is one of the rare MIS 3 sediment sections preserved in glaciated areas in northern Fennoscandia and in the Arctic region. The stratigraphy described in this article has a consistent chronology, and the stratigraphy and proxies show clear, continuous succession. This suggests our section represent a whole stadial-interstadial-stadial cycle starting from the Middle Weichselian glacial phase of MIS 4 and continuing to the deposits of whole ice free period of MIS 3 before ending to the till of the Late Weichselian stadial. The section is finally covered by the Holocene sediments and vegetation. Particularly, the full biostratigraphical data describe for the first time the full vegetation succession of MIS 3 ice free phase in northern Fennoscandia, at the central part of SIS.

The dominance of small *Fragilaria* spp. diatoms at the bottom of MIS 3 sediment unit suggests a formation of a glaciolacustrine environment. These taxa are a characteristic for cold postglacial environments with prevailing erosion and deposition of sandy sediments brought from the open surrounding areas^44^,^45^,^46^. In general, afterwards the bryophyte taxa and diatoms indicate dynamic deposition, rapidly changing environmental conditions with the influence of possible spring waters and occasional flooding because of melt-water. The sedimentary record of the section supported by the diatom succession indicate climate change that resembles those prevailed generally in northern Fennoscandia during the last deglaciation at the end of MIS 2 and Holocene^41^,^47^.

Occasionally macroscopic plant remains derived from the lake/wetland sections were quite degraded possibly due to fluvial activity, and this sometimes hampered fully reliable species-level identification. In any case, combined plant macrofossil and pollen data indicate relatively diverse plant community around the lake already from the beginning of the organic sediment section. For instance following taxa were detected: *Callitriche* cf. *cophocarpa* seed, supplemented by *Callitriche*, *Hippuri*s, *Myriophyllum*, *Potamogeton, Moneses uniflora* and *Filipendula* pollen coming from local pollen sources^15^, *Comarun palustre* seeds, *Montia fontana* seeds and tree *Betula* seeds and catkin scales. *Pinus* needle found from the top part of the section confirmed the local presence of conifers, yet the presence of conifer bark was suspected already earlier. This combination of taxa indicates boreal rather than subarctic environmental conditions, currently none of these species occur above tree line^48^.

The amount of wood was conspicuous in the top layers. A set of thin-sections were prepared from some of the wood pieces to investigate the cell structures but these examples represented deciduous wood not conifers. However, some bulk peat samples from the same sediment section were previously analysed for ancient DNA composition and then pine DNA was detected^49^. Also in these first try-out samples *Pinus* pollen values were low, *Betula* pollen values were high and no conifer macrofossils were found. Based on modern distribution ranges of species such as *Callitriche, Sagittaria* and *Nymphaea* from Kaarreoja samples it seems that the minimum July temperature was at least 14.4 °C^31^,^50^,^51^. The bryophyte species *Polytrichum commune* is a typical conifer forest species. In one top sample it formed 10% proportion of all plant remains. This supports our interpretation of forested landscape. Previously, presence of boreal species, based on macroscopic remains, dated to MIS 3 have been reported by Väliranta *et al*.^26^ but otherwise, in principal, open subarctic MIS 3 conditions, possibly with some mountain birch individuals, has been suggested^3^,^4^,^5^,^6^,^12^,^52^. Thus, our data are striking – for the first time there is a clear evidence of MIS 3 presence of pine and establishment of birch forest in Fennoscandian north. And the implication of these data are that also MIS 3 phase shows a climate and environmental succession pattern typical for earlier interstadial MIS 5c and interglacials in northern Fennoscandia^48^.

Earlier studies have not recognised this area to be forested during MIS 3. For example, MIS 3 interstadials have been reported from northern Finland at Sokli^2^,^3^,^5^,^24^ and Petäjäselkä^4^ and from northern Sweden, at Riipiharju^12^ and possibly at Rissejauratj^46^ (Fig. 1). Despite the pine pollen percentages were relatively high, up to 28%, in Sokli site^2^,^3^, accompanied by 18–37% proportion of *Betula* pollen, these pollen assemblages were interpreted to represent shrub tundra environment. High values of *Betula* (c. 30%) and *Pinus* (c. 30–40%) at Petäjäselkä^4^ were originally interpreted to represent tree line conditions with individual scattered birch trees. This interpretation was later slightly changed and more boreal conditions were suggested based on macrofossil data^26^. It should be noted that Petäjäselkä section was only a thin peat lens between till beds, not a full stratigraphic section. MIS 3 record from Riipiharju (Riipiharju II) shows apparent hiatuses in the sediment section^12^. *Betula* values vary considerably from 1 to 65% while *Pinus* percentages remain low 0–8% and the interpretation was an open birch forest^12^. The absence of termophilous species in Riipiharju^12^ and Sokli^2^,^3^ records may reflect the fact that these records represent different MIS3 phases. The individual findings of *Quercus* and *Carpinus* in Petäjäselkä record were interpreted as a probable result of the long-distance transportation^4^. However, interpretation of fluvial environment pollen assemblages is less straightforward than assemblages derived from lacustrine environments, as the fluvial activity may increase the proportion of long-distance transported or re-deposited pollen^53^,^54^. But, in Kaarreoja the amount of broken and crumpled pollen was low in all samples, thus re-deposition was not likely.

Moreover, it should be bear in mind that sedimentology of these sections differ: the lacustrine sediment records in Sokli and Riipiharju consist of mostly mineral material whereas Petäjäselkä peaty profile compares to Kaarreoja peaty section 235–232 cm (Fig. 3). All in all, the Kaarreoja deposition environment varied more through time than in the other MIS 3 sites. The smaller proportion of *Pinus* in Kaarreoja when compared to the Petäjäselkä and Sokli may be explained 1) more northern location (100 km), 2) higher altitude c. 351 m vs c. 270 m a.s.l. 3) taphonomical processes related to fluvial environment.

It can be speculated why previous studies have not come to a conclusion of boreal conditions. First of all, earlier studies may have suffered from too fragmented layers which do not cover the whole interstadial but perhaps only had an early part of the interstadial, when the trees had not yet returned from their glacial refugia. Secondly, the preserved MIS 3 sediment sections have often been very minerogenic^55^,^56^,^57^,^58^, not lake or wetland depositions which would preserve plant remains, macrofossils and pollen, better. In any case when a proper age-depth model is lacking, it is not possible to calculate pollen accumulation rates and this has hindered using pollen data to interpret if the environment was forested or not. Our data suggest that even a small proportion of conifer pollen in a wetland material like in Kaarreoja could indicate local presence of a species. We propose that there might be room for environmental and climate re-consideration since many of the previous MIS3 studies report of quite high birch and pine pollen proportions^4^,^14^,^24^, thus probably also these reflect actual presence of pine.

In conclusion, this study showed that MIS 3 interstadial probably resembled a climate and environmental development which was reconstructed for MIS 5c interstadial for Finland^47^,^59^ but the record also show features typical for Eemian and Holocene interglacial developments^15^,^60^,^61^. Our data agree with insolation reconstructions^14^ and correspond with the suggestion that glacial stadial–interstadial and interglacial stages have been much more dynamic than previously thought^51^. The July mean temperature c. 14.4 °C during MIS 3 in Kaarreoja actually corresponds well to present-day lowland mean temperature in the Kaarreoja region, northernmost Finland.

Our data suggest that boreal forested environment prevailed during the MIS 3 in northern Fennoscandia. Frequent tree birch seed findings and pine needle require the local growth of forest. July temperature was at least 14.4 °C based on presence of aquatic species *Sagittaria sagitifolia*. Also other aquatic species suggest July temperatures at least 13.65 °C. Such temperatures are characteristic for present-day July at middle to northern boreal Finland. It seems that in sediment records representing mire and fluvial deposition environments even a small proportion of pine pollen might indicate actual presence of the species. Our results radically change the understanding of climatic conditions during the ice-free interstadial stages of last glaciations.

## Methods

**Study site** in Kaarreoja (68.65°N, 25.63°E, c. 350 m a.s.l.) is located in the Lemmenjoki area, northern Finland (Fig. 1). The study site is currently dominated by semi-open mountain birch (*Betula pubescence, ssp. czerepanovii*) forests. Pine (*Pinus sylvestris*) grows sparsely on valleys and valley sides. Upper in topography, at the level 380–400 m a.s.l. forests disappear forming open fell tops. The northern limit of the spruce (*Picea abies*) forests runs c. 50 km to the south of the study site. Peatlands support dwarf shrub vegetation such as *Betula nana* and willows (*Salix* spp.) in lowland areas. The bedrock is Palaeoproterozoic granulite belt with some gabbro veins^62^. Late Quaternary deposits comprising glacial till and glaciofluvial or fluvial sediments are dominant as a surficial sediment cover. Modern river Kaarreoja is very narrow (1–3 m) and shallow (c. 0.5 m in depth). The area is located on the northern side of the last ice divide zone of the Scandinavian Ice sheet with ice flow in the direction SSW-NNE^1^,^13^. Annual average precipitation is 500–550 mm and annual mean temperature −2–−1 °C, July mean temperature is ca. 11–12 °C. The snow cover lasts ca. 6–7 months from November to May.

**Sedimentological observations and sampling** was carried out using excavated sections in the river valley of Kaarreoja. The sediment sections were logged and the results described in the stratigraphy log (Fig. 2) using lithofacies classification accordingly^63^. Continuous sediment samples were collected from organic sediment deposit using steel boxes (55 × 10 × 5 cm) and about one kilogram samples into plastic bags for the grain size analyses. Grain size analyses were done using dry sieving and Sedigraph analyser in Labtium laboratory in Finland.

**Dating samples** were taken both from the peat (for ^14^C dating) and the stratified sediments under organic sediments (for OSL dating). For the radiocarbon dating both bulk peat samples as well as wood pieces were collected from the section 29 (Fig. 2). The OSL samples were collected from the sections 29 and 30 into black plastic tubes c. 7 cm in diameter and 20 cm in length avoiding the sun-light contamination. Immediately after the sampling the tubes were covered with aluminium folio and black plastic bags for avoiding any contamination during storing and transportation. The dating analysis (Hel-TL04274 and Hel-TL04275) using quartz grains were done based on the SAR protocol described by Murray and Wintle^64^ in the Dating laboratory of the University of Helsinki, Finland. The grain size of the quartz grains used in the analysis range 0.210–0.297 mm. The assumption was that the samples were fully saturated with water (water content ~20%) most of their burial time. The water content correlation equation for the gamma dose rates^65^ was applied to the rates in a water-saturated situation. ^14^C dating was done for the bulk sample Tln3277 in the Institute of Geology at Tallinn University of Technology, Estonia and for the wood pieces (Hela-2693) by the radiocarbon determinations using Accelerator Mass Spectrometric (AMS) method^66^ in the Dating laboratory of the University of Helsinki, Finland.

**Pollen analyses** were done from peat and organic gyttja samples. Continuous, 55 cm long peat-sediment section was analyzed with 1–5 cm interval for pollen content and the total pollen count (∑P) was calculated from the percentage of terrestrial vascular plants (trees, shrubs, dwarf shrubs and herbs). A total of 13 samples were analysed for pollen content. One cm^3^ of each sample were extracted for analysis and processed using heavy liquids modified from^67^ and from Zabenskie & Gajewski (http://www.lpc.uottawa.ca/resources/pollen-heavyliquid.html). Lithium heteropolytungstate solution (LST) was used instead of sodium polytungstate (SPT) and without HF treatment. A minimum of 500 terrestrial vascular pollen grains and spores were counted with 400x magnification. Aquatic species, bryophyte spores and green algae Pediastrum were excluded from the pollen sum. Spores were calculated from the total pollen count and spores (∑P + spores). *Betula* tree was separated from the *Betula nana* based on the grain diameter and morphological characteristics (height of the pore)^68^,^69^,^70^. The pollen diagram was compiled using the Tilia software^71^. Loss-of-ignition (LOI) analysis was carried out by a standard SFS-EN 15935, igniting the dried samples for 2 h at 550 °C.

**Plant macrofossil analyses** were obtained from c. 30 cm^3^ subsamples and altogether 31 samples were analysed. The subsamples were rinsed under running water using a 140 μm sieve. The material retained on the sieve was systematically examined under a stereomicroscope. No chemical treatment was necessary.

### Plant-based July temperature reconstructions

We used macrofossils and aquatic pollen to quantitatively reconstruct July temperature. Macrofossil- derived reconstructions are not continuous in the same way as pollen reconstructions but they are based on the presence of indicator species and often samples do not contain any remains that may be used to infer temperature. However, presence of macrofossils of indicator species provide reliable material for local environmental reconstructions^31^,^72^. Moreover, like macroscopic plant remains also aquatic pollen is only locally dispersed^73^ and can be applied in a similar manner as macrofossil data, yet these data are typically neglected.

Sample-specific error estimations as with pollen reconstructions are not possible with the macrofossil (and aquatic pollen) indicator species method, which is based on presence of a taxon. In this study the reconstructed species-specific mean July temperature is based on the median of modern mean July temperature observations at grid cells containing individual occurrences at the modern northern species limit (Supplementary Fig. S3, Table S2). The median value incorporates July temperature values of individual outlying occurrences including those which may also be situated in unusually favorable microhabitats with an ideal microclimate and/or an ideal combination of secondary ecologically significant environmental factors. Thus, it may be considered that the derived July temperature values are conservative, i.e. low, rather than being overestimations. The extent of the largest possible July temperature overestimation can be derived from the differences between the “median observed” and the “lowest observed” July temperature at the northern distribution limit for each species. These median-to-lowest differences typically vary around 0.50 °C^31^ (Supplementary Table S2).

A species-specific modern geospatial plant distribution data-set (http://www.luomus.fi/kasviatlas)^50^ covers the whole of Finland, and long-term meteorological climate data are available^48^ (Supplementary Fig. S3, Table S2). Thus, the plant-distribution data, which is based on continuous botanical surveys, can be correlated to climate variables.

Relatively few taxa were identified to species level. However, especially two of these species have important temperature indication value. These species are: *Nymphaea* (only two species occur in Finland), *Callitriche cophocarpa and Sagittaria sagitifolia*. The lowest July temperature limit ( °C) where these species occur today is indicated in brackets (see also Supplementary Table S2). *Nymphaea* (13.49 °C), *Callitriche cophocarpa* (13.65 °C) and *Sagittaria sagitifolia* (14.43 °C).

**Diatom analysis** of sediment samples followed the technique presented by Battarbee (1986)^74^. Slides for microscopic analysis were prepared using “Naphrax” as amounting medium. Slides were studied under a light microscope “Nicon” (magnification x1000). Diatom species were identified mainly following the taxonomic work of Krammer and Lange-Bertalot^39^. Some diatoms were identified only to genus level due to bad condition of valves or girdle view of diatoms (*Fragilaria* spp.). The succession of the most frequent and ecologically important taxa is presented as percentages of the total sum of the identified diatoms. In order to describe the palaeoecological conditions of the water basin, diatom species were classified into ecological groups^36^,^37^,^38^ which are based on habitat requirements: 1) planktonic and free-floating taxa; 2) benthic and bottom-living taxa and 3) epiphytic and other littoral taxa attached to various types of surfaces. The percentage composition of each diatom species was calculated out of total diatom sum in the sample. “TILIA“^71^ аnd “TILIA&GRAPH” computer softwares^75^ were used to create diagrams.

## Additional Information

**How to cite this article**: Sarala, P. *et al*. First physical evidence for forested environment in the Arctic during MIS 3. *Sci. Rep.* **6**, 29054; doi: 10.1038/srep29054 (2016).

## Supplementary Material

Supplementary Information
