# Supplementary material for: First physical evidence for forested environment in the Arctic during MIS 3
Source: Sci Rep. 2016 Jul 1;6:29054. doi: 10.1038/srep29054 (PMC4929562; doi:10.1038/srep29054)
Supplement: Supplementary Information [file srep29054-s1.pdf]

SUPPLEMENTARY INFORMATION TO

'FIRST PHYSICAL EVIDENCE FOR FORESTED ENVIRONMENT IN THE ARCTIC DURING MIS 3'

Pertti Sarala[1*], Minna Väliranta[2], Tiina Eskola[3] and Giedré Vaikutiené[4]

[1] Geological Survey of Finland, P.O. Box 77, FI-96101 Rovaniemi, Finland

[2] Department of Environmental Sciences, P.O. Box 65, FI-00014 University of Helsinki, Finland

[3] Oulu Mining School, P.O. Box 3000, FI-90014 University of Oulu, Finland

[4] Vilnius University, Čiurlionio 21/27, LT-03101 Vilnius, Lithuania

[*] Corresponding author E-mail: pertti.sarala@gtk.fi

Grain size analyses

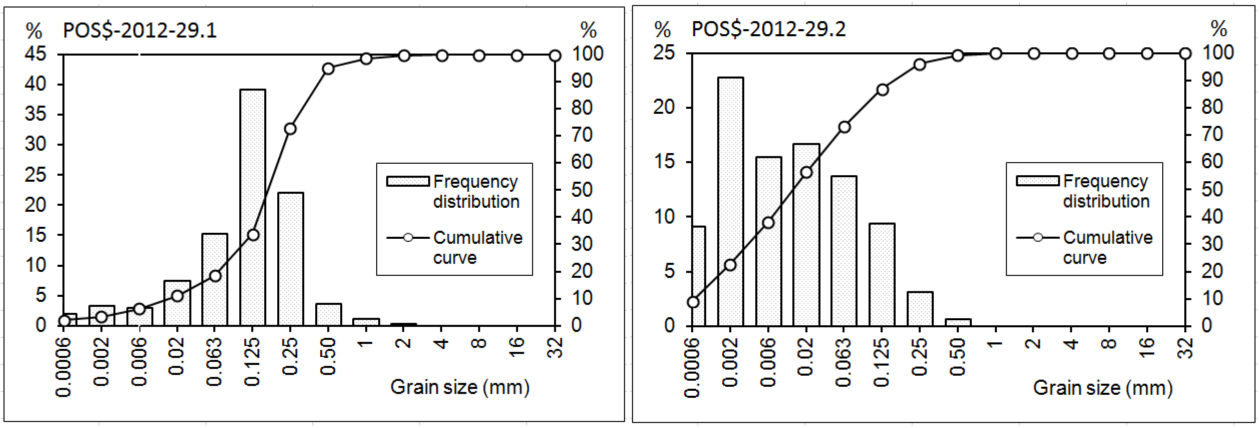

Fig. S1. Grain size analyses of the sediment samples in the section POS$-2012-29. 29.1 is representing the layer (Unit 3) of which the OSL dating was done and 29.2 is representing the lacustric sediment in the Unit 4. Grain size analyses were done using dry sieving and Sedigraph analyser in Labtium laboratory in Finland.

OSL dating data

The number of aliquots that was tested was 10 to the sample Hel-TL04274 from the section POS$-2012-29.1 and 8 to the sample Hel-TL04275 from the section POS$-2012-30.1. Two aliquots of the first sample and three of the second sample were rejected due to too high instability or too old age. The rest, i.e. 8 in the first sample and 5 in the second sample, were considered as consistent (within 2 sigma) and reliable for dating purpose. The finite mixture model was used to define ages.

A

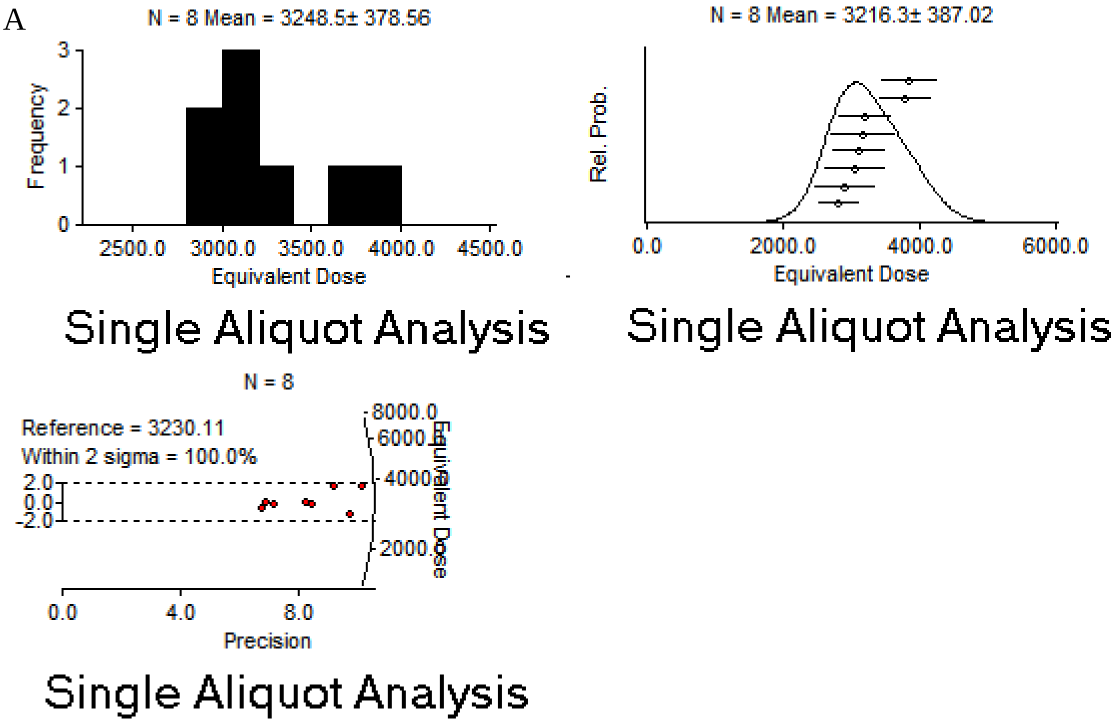

B

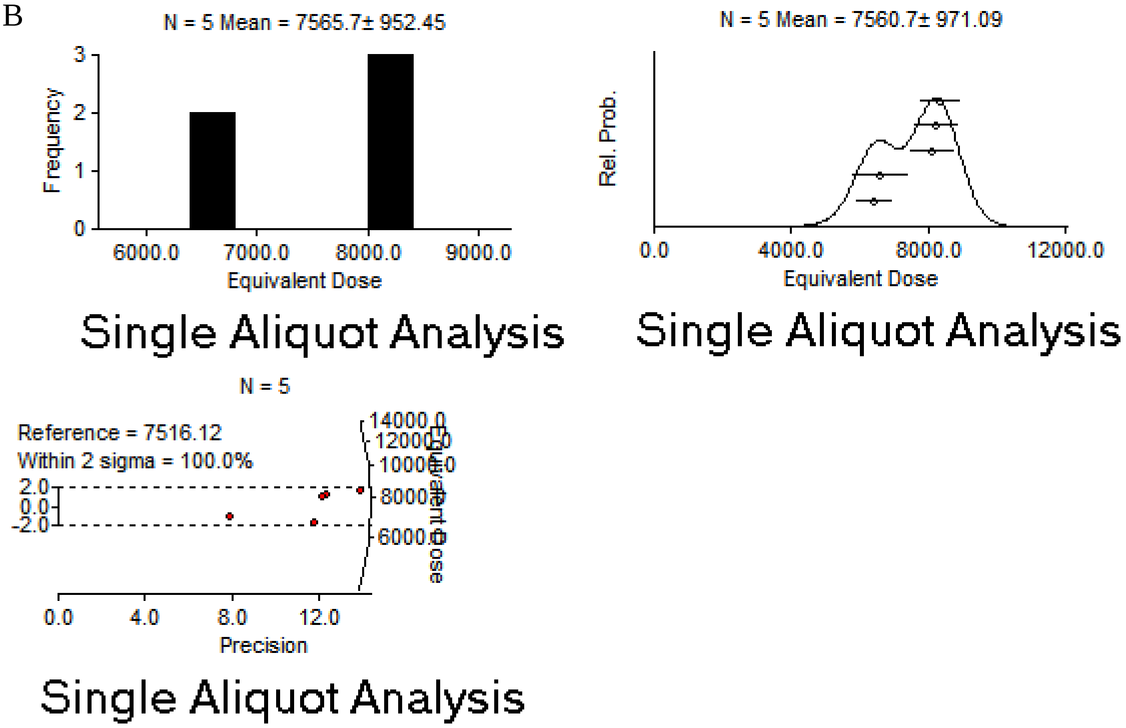

Fig. S2. Aliquot analysis of the OSL samples A) Hel-TL04274 from the section POS$-2012-29.1 and B) Hel-TL04275 from the section POS$-2012-30.1. The grain size of the quartz grains used in the analysis range 0.210-0.297 mm.

Plant-based reconstructed July temperature

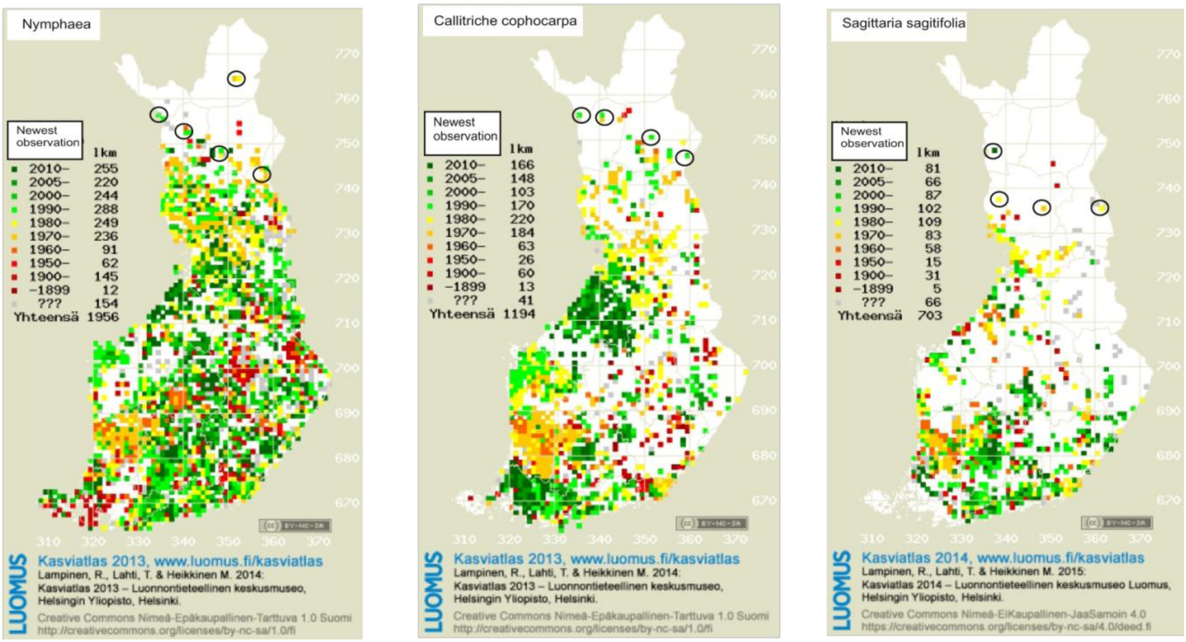

Fig. S3. This figure illustrates how the plant-based reconstructed July temperature was achieved. First a set of species observations that are located at the northernmost border of the current distribution range (circles) was selected. Only 1970-2000 (light green and yellow symbols) observations were accounted because this corresponds to the used period of the meteorological data. In Lampinen & Lahti (2013) data base all plant observations have x and y coordinates, thus all observations can be linked to the meteorological data calculated for the same grid cells (Venäläinen et al. 2000). These combined data were used to estimate the lowest mean July temperature range where the species currently occurs and subsequently to reconstruct past July temperatures.

TABLES

Table S1. The list of OSL results from the Kaarreoja sediments.

Lab code	Sample code	Site	Depth (m)	Dose rate, Gy/ka	Equivalent dose D_e, Gy	Number of aliquots (n)	Age (ka)
Hel-TL04274	POS$-2012-29.1	Kaarreoja	2.8	1.76±0.31	91±11	8	52±12
Hel-TL04275	POS$-2012-30.1	Kaarreoja	6	1.61±0.25	215±28	5	130±28

Table S2. Modern mean July temperature values at the modern species distribution limits. We used Venäläinen et al. (2005) meteorological data to interpolate 10x10 km grid cell-specific mean July temperatures. This was done respectively for each indicator species using several grid cells containing species occurrences along the current (observations 1970-2000) distribution boundary (Lampinen & Lahti 2013). Bold values are the medians of the sampled mean July temperature values and this species-specific July temperature limit is used in quantitative reconstructions. The lowest species specific mean July temperature observation is shown in Italics, the highest observed temperatures are underlined.

	N:E coordinates	July mean	July mean minimum	July mean maximum	July lowest minimum	July highest maximum
Nymphaea						
	7565000:3345000	13.37	7.95	19.01	5.5	23.8
	7525000:3415000	13.61	7.77	19.44	4.5	23.7
	7645000:3515000	13.49	7.3	20.18	4.7	26.1
	7545000:3525000	*13.23*	6.94	19.62	4.3	24.1
	7445000:3585000	*13.98*	8.05	19.58	5.1	24.4
		**13.49**				
Callitriche cophocarpa						
	7555000:3355000	*13.5*	7.92	19.2	5.2	24.1
	7555000:3405000	13.46	7.77	19.28	4.5	23.9
	7505000:3515000	13.79	7.76	19.71	4.9	24.4
	7475000:3555000	13.89	7.71	19.67	4.6	24.4
		**13.65**				
Sagittaria sagitifolia						
	7485000:3375000	*14.08*	8.5	19.63	7.7	23.6
	7375000:3385000	15.15	9.91	20.36	7.2	25
	7355000:3485000	14.49	8.94	19.89	5	25
	7355000:3615000	14.36	8.52	19.77	5.5	24.53
		**14.43**				

References

Venäläinen, A. et al. A basic Finnish climate data set 1961–2000 – descriptions and illustrations. *Finnish Meteorological Institute, Meteor. Rep.* **2005:5**, p. 27 (2005).

Lampinen, R. & Lahti, T. *Kasviatlas*. Helsingin Yliopisto, Luonnontieteellinen keskusmuseo, Helsinki. (2013).
